# The Functional Form of the Relationship between Body Height, Body Mass Index and Change of Direction Speed, Agility in Elite Female Basketball and Handball Players

**DOI:** 10.3390/ijerph192215038

**Published:** 2022-11-15

**Authors:** Marek Popowczak, Pavol Horička, Jaromir Šimonek, Jarosław Domaradzki

**Affiliations:** 1 Faculty of Physical Education and Sport, Wroclaw University of Health and Sport Sciences, I.J. Paderewskiego 35, 51-612 Wroclaw, Poland; 2 Department of Physical Education and Sport, Constantine the Philosopher University in Nitra, Tr. A. Hlinku 1, 94901 Nitra, Slovakia

**Keywords:** competitive sport, cognitive motor skills, team sport, performance analysis

## Abstract

The aim of this study was to assess the functional form of the relationship between two anthropometric measurements—body height (BH) and body mass index (BMI)—and two motor abilities—change of direction speed (CODS) and agility (AG)—in female elite basketball and handball players. It was hypothesized that BH and BMI might be significantly associated with AG and CODS. Two scenarios of the Five-Time Shuttle Run to Gates test (planned and unplanned) were used to evaluate the CODS and AG. Two forms of models were built to assess functional forms of the relationships between CODS and AG vs. BH and BMI: simple linear regression and binomial curvilinear regression for each type of team sport. The results confirmed the relationships between both anthropometric measurements and motor abilities only in HB, whereas in BB only a significant relationship was noted between BH and AG. Moreover, two curvilinear functional forms of the relationship were identified: inverted L-shape and inverted U-shape. Therefore, it was concluded that the first form of function indicated an initially proportional relationship between anthropometric measurement and motor test results and plateau after reaching a certain value of the BH or BMI. Similarly, the second form of function indicated the peak value of the BH or BMI which is threshold for the value of the anthropometric measurement when a progressive effect in the functional feature occurs.

## 1. Introduction

Agility (AG) is predefined as a rapid, whole-body movement with a change of velocity or direction in response to a stimulus [1,2]. This ability is a parameter closely associated with effective movement, and this is considered to be a key component of the performance of athletes of team sports [3]. It comprises two key components: change of direction speed (CODS) and perceptual and decision-making factors. Therefore, a high level of agility is not strongly dependent on acceleration and sprinting speed alone, but also on the ability to coordinate movement, technical skill, anticipate other player’s actions, visually scan the environment, and react to the visual cues of one’s opponents in order to provide a quick and appropriate response [1,3,4,5]. The anthropometric parameters also play a significant role in the agility results, for example, due to the importance of body dimensions in the results of motor tests, i.e., body height, leg length, or body mass index [6,7,8]

CODS on the other hand, is speed in the ability to accelerate, stop quickly, turn, or change direction and accelerate again. CODS involves some form of quick, directional displacement of the center of mass (COM) relative to the base of support (BOS) [3]. The CODS and AG should be investigated separately [1,4] because CODS does not require a response to a stimulus and is usually classified as a pre-planned and closed skill [9,10]. The motor and anthropometric parameters will be primarily responsible for the result of the COD tests [11]. Both abilities, AG and CODS, are important factors of athletes’ play in basketball and handball.

The importance of body dimensions such as body height or leg length in the results of motor tests determining the components of agility has been demonstrated in numerous studies [7,12,13]. Lower body height in shorter players, and at the same time lower COM position, is important for getting better results in CODS and agility tests [8,14]. To the authors’ knowledge, it is not known whether the relationship between these anthropometric parameters and CODS and agility takes the form of linear dependencies. Another anthropometric index that proves good body composition is BMI. It indirectly indicates the amount of adipose tissue [15,16].

Based on previous studies, it cannot be unequivocally said that there is a significant relationship between BMI and CODS or an agility task in a professional female basketball and handball player. In the previous studies of team sports athletes, first of all, no significant relationship was found between the BMI and results of AG and CODS tests [17,18,19,20]; however, significant relationships were noted in male soccer players [7] aged 14–18 (BMI vs. AG, r from −283 to −0.245) or in male professional football players (BMI vs. AG, r = 0.76) [21]. On the other hand, it is important in handball to have a high fat-free mass, which can result in higher BMI values for being successful in sports [22]. Therefore, various forms of functional relationships between BMI vs. CODS and AG, which may be curvilinear, should be sought.

There is much evidence in biological studies and studies concerning motor development that has shown curvilinear (second or higher term) rather than rectilinear relationships between physical features or motor ability [23,24,25,26]. The analysis of simple relationships (correlation and regression) often showed weak relations between coordination abilities and anthropometric measurements in athletes. The players’ observations and the collected empirical material indicate that the relationship between morphological and motor features is not rectilinear. It does not increase monotonic but increases exponentially. Within a certain interval of the independent variable, the dependent variable is directly proportionally dependent, but outside this interval, the changes increase or decrease. In this case, the entire relationship is better described by a polynomial (second or third degree) function. Some studies even indicate curvilinear relationships (especially in relation to sport).

Numerous studies have attempted to assess CODS and AG under training conditions; however, few of these are based on the same movement pattern [27,28], whereas Popowczak, Cichy, Rokita and Domaradzki [8] noted that it is important to standardize patterns in planned and unplanned tasks. This allowed for the determination of the motor component (CODS) and the perceptual-cognitive component measured in an agility task based on a similar movement pattern. In addition, it is important that the tests used for basketball players or handball players are based on a specific ‘stop-and-go’ scenario [8,29] and reflect a distinctive change of direction movement required in basketball and handball.

Current studies on associations between body and agility measurements are limited mainly to the assessment of the simple relationships or moderation role of the anthropometric measurements in coordination abilities analyzes. There is a lack of studies searching for the mathematical functions (functional forms) which are fitted the best to the relationship between the body height, weight, or body mass index and COD and AG. To the best of the authors’ knowledge, there are no studies on the shape (functional forms of such relationships) of the relationship between basic physical characteristics (BH and BMI) and CODS and AG. Moreover, most research has focused on males, and less information is available to describe the appropriate predictors in elite female basketball and handball players [17,30]. Therefore, the aim of the study was to assess the functional form of the relationship between two anthropometric measurements—body height (BH) and body mass index (BMI)—and two motor abilities—change of direction speed (CODS) and agility (AG)—in female elite basketball (BB) and handball (HB) players. The authors hypothesized that BH and BMI might be significantly associated with the results of CODS and AG. Moreover, the relationships between anthropometric parameters and the results of motor ability might be curvilinear.

## 2. Materials and Methods

### 2.1. Participants

The study group consisted of 31 elite female athletes, including 12 BB (age: 24.98 ± 3.38 years; body mass: 74.38 ± 8.54 kg) and 19 HB (age: 27.34 ± 4.68 years; body mass: 71.13 ± 8.35 kg). All BB belonged to the same team, competing in two basketball leagues—Energa Basket League Women (1st League in Poland) and EuroCup Women—in the 2018–19 season. All the HB were on the same team, competing in the handball league PGNiG Women’s SuperLeague (1st League in Poland) and the EHF Women’s Cup in the 2018–19 session. Access to the players in this study represented a sample of convenience (which is a limitation of this study). The criterion for inclusion was participation in the previous season in the 1st National League as a starter player and preparation for participation in EuroCup for the next season. The study was conducted one week following a preparatory period.

This study was approved by the Research Bioethics Committee of the Faculty Senate of the Wroclaw University of Health and Sport Sciences (reference number: USPE-2013-06-07) and conducted in accordance with the ethical principles for medical research involving human subjects contained in the Declaration of Helsinki by the World Medical Association. The study also met the ‘Ethical standards in sport and exercise science research’ [31]. All participants were asked to provide written informed consent prior to the study, and the purpose and characteristics of the research were explained.

### 2.2. Measurements

**Figure 1:**
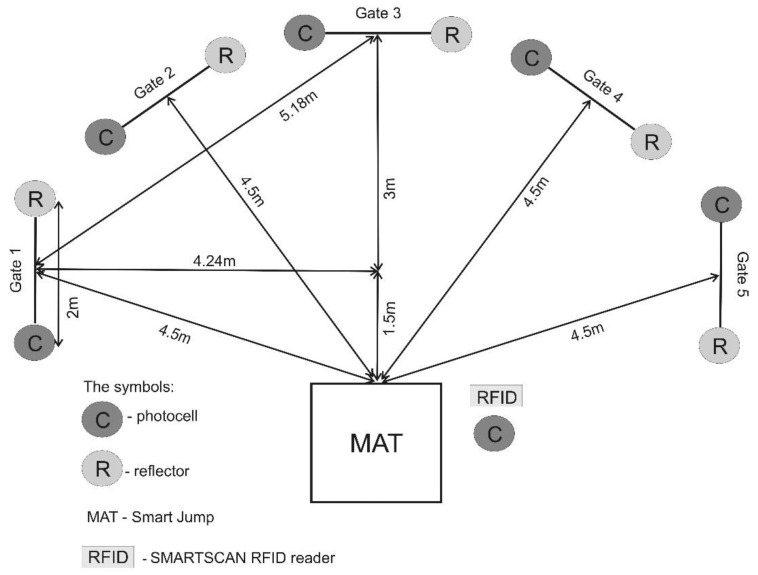
‘The Five-Time Shuttle Run to Gates’ Test [4], reprinted with the author’s permission.

All the tests were carried out in the sports hall where the participants usually played and trained. Therefore, each participant was able to perform the test at the maximum level. The tests were carried out in a single day. The height of the athletes was measured with a GPM 101 anthropometer (DKSH, Zurich, Switzerland), which has an accuracy of 1 mm. The participant’s body mass index was measured when they were modestly dressed and without shoes using an InBody 230 system (Tanita Corp., Tokyo, Japan). Before starting to measure motor ability, the participants underwent a standardized 15 min warm-up procedure. The participants were then familiarized with the ‘Five-Time Shuttle Run to Gates’ test. Then, in the testing session, they performed the test to determine CODS and AG. A Fusion Smart Speed system (Fusion Sport, Coopers Plains, QLD, Australia) was used during the test. The system consisted of photocells (with an infrared transmitter) and light reflectors, a Smart Jump mat integrated with a photocell, an RFID for identifying athletes, and computer software (Figure 1). The total time of the test was reported to the nearest 0.001 s. The test data (with the participant’s name) were saved in a PDA (HP iPAQ 112) compatible with the Fusion Smart Speed system. All data have been exported to a Microsoft 365 Excel spreadsheet (version 2017) and statistical software.

‘The Five-Time Shuttle Run to Gates’ test measures the time of repeated ‘stop-and-go’ directional changes to a light signal. The Fusion Smart Speed System application was used for the fixed (pre-planned) or random selection of a gate (non-planned), in accordance with the procedures proposed by Popowczak, Rokita, Struzik, Cichy, Dudkowski, and Chmura [32]. In both the planned and non-planned tests, the participant had to run the distance from the mat to the gate’s line (4.5 m) and return to the mat five times (placed between photocells with reflectors 2 m apart) as quickly as possible. The mat had integrated photocells. The layout of the gates, mat, and RFID reader in the ‘Five-Time Shuttle Run to Gates’ test is illustrated in Figure 1. The participant, once they touched the mat with both feet, received a light signal indicating the gate they should run to. The start was not delayed gates. In the non-planned ‘Five-Time Shuttle Run to Gates’ Test, which measures AG, the participants ran to the gates in a random order for all participants. In the pre-planned ‘Five-Time Shuttle Run to Gates’ test, which measures CODS, the participants ran to the gates in a set order (1-2-3-4-5), which was uniform for all participants. The AG and CODS tests were repeated twice. Each participant rested for 5 min between repetitions in each test. The best results (total time) of the run (AG and CODS) were used in the analysis. In both tests, all participants covered the same distance.

The reliability analysis of the results ‘The Five-Time Shuttle Run’ test indicated statistically significant reliability for both CODS (Cronbach’s alpha: 0.898) and AG (Cronbach’s alpha: 0.865).

### 2.3. Statistical Procedures

The Shapiro–Wilk test was used to evaluate the normality of the distribution of the continuous variables. The variables showed a normal distribution with *p* > 0.05. Descriptive statistics as well as comparisons between two disciplines were presented elsewhere [8,33] as means, standard deviations, 95% confidence intervals (CI), ANOVA, and partial correlations.

The associations between outcome variables: CODS and AG which were dependent variables (DV) in models, and independent variables (IV)—anthropometric measurements: BH and BMI. Two forms of regression models were built to assess the functional forms of the relationships: simple linear regression and binomial curvilinear regression. In all models, coefficient and squared coefficients in regression function, Constant, R-squared (R^2^)—coefficient of determination and *p*-value were calculated to evaluate the goodness-of-fit received model. Separate models were built for each type of team sport.

The first step was to calculate simple regression equations with slopes and intercepts (b0. b1). The second step was to study the curvilinear relationship between DV and IV using a quadratic function. The value of the maximum or minimum of the quadratic function was computed too. Based on the equation y = ax^2^ + bx + c the following formula was used to calculate the peak value: max =—b/2a. The results were plotted and presented as scatterplots.

Statistical significance was set at α = 0.05. We used Statistica version 13.0 (StatSoft Polska, Cracow, Poland 2022) for data analysis.

## 3. Results

Descriptive statistics, Pearson’s product moment correlation coefficients, and comparisons, as mentioned above, were presented elsewhere [8]. Based on our research, it was noticed that the BB differed (*p* = 0.033) in height from the HB (Table 1). BB had a BH = 181.67 cm and were 5.9 cm higher than HB. However, the elite athletes of both team sports presented similar BMI values. HB achieved better results in motor tests. They were faster (*p* = 0.041) in the CODS test by 0.62 s. In the agility test, HB were faster by 0.83 s than BB (*p* = 0.014). Based on these results, the next stages of the analysis were carried out, taking into account the type of sport. Here, we presented in depth analyses of the functional forms in relationships between anthropometric measurements and AG and CODS, regarding the type of the team sport.

Firstly, two models of the relationships between CODS vs. BH, BMI and AG vs. BH, BMI for each type of team sport were calculated. This step gave a general overview and insight into the significance and model’s fit studying pattern of the relationship between anthropometric measurements and functional features. It allowed for the evaluation of the importance of BH and BMI in the explanation of COD and AG in elite BB and HB (Table 2).

Thus, two comparative analyses were used. In the first model (simple linear regression), only BH or BMI were analyzed. In the second model (binomial curvilinear regression was used), BH^2^ and BMI^2^ were included, respectively. This procedure allowed us to check if adding higher order terms for anthropometric measurements did improve the model fit. The results are displayed in Table 2.

The results clearly indicated that there were differences in functional forms between disciplines, as well as in patterns in the relationship between each anthropometric measurement and coordination ability (Table 2).

**Figure 2:**
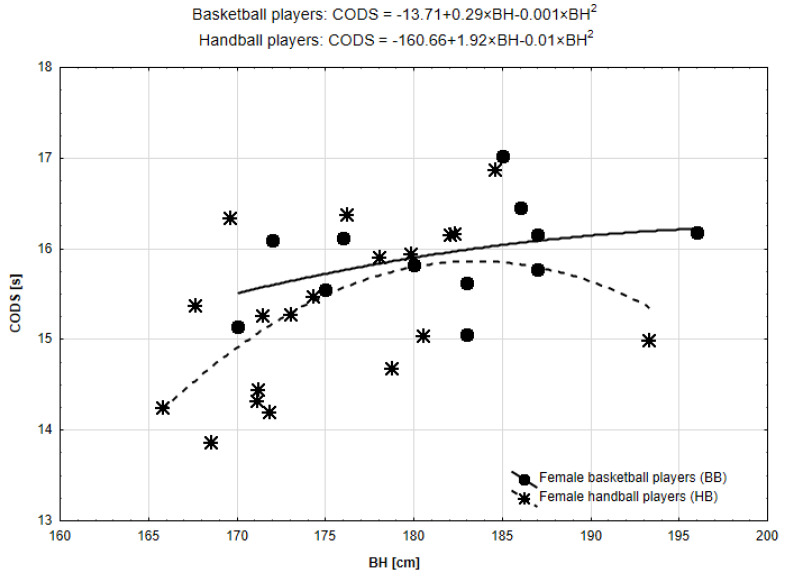
Functional forms of the relationships between body height (BH) and change of direction speed (CODS) in BB and HB.

Taking into account the relationship between BH and CODS, in the case of BB, both models were similar (both R^2^ = 0.15, non-significant). In the case of HB, the quadratic function was better fitted and statistically significant (R^2^ = 0.32, *p* = 0.045). Figure 2 presented scatterplots. The curve for HB lies below the line for BB, which confirms generally better results in the CODS trial of the handball players. However, the parabolic type of the line suggested a tendency to constantly worsen the results, up to a certain level of body height (peak value of the parabola) and a definite improvement above that value of the body height. The calculated peak of the body height at 183 cm was the threshold for improvement of the CODS results.

**Figure 3:**
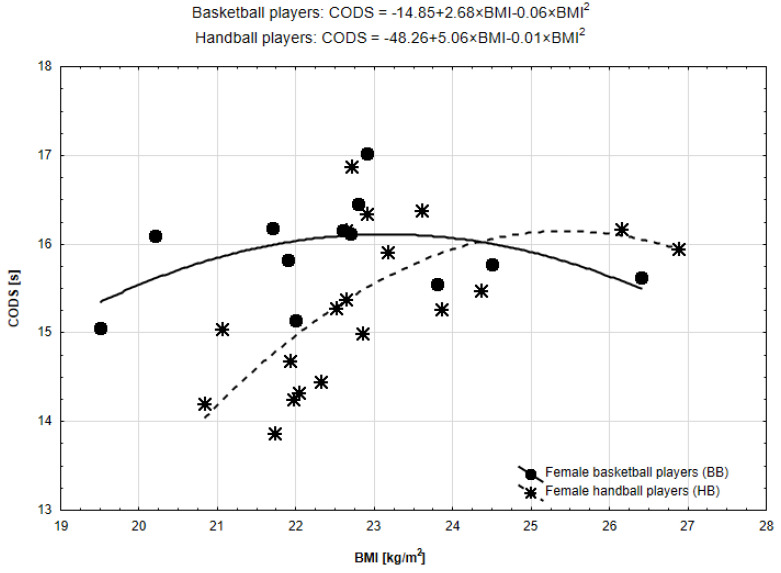
Functional forms of the relationships between BMI and CODS in BB and HB.

Taking into account the relationship between BMI and CODS, in both cases (BB and HB, Figure 3), a quadratic function was better fitted than the linear one in both team sports but statistically significant only in HB (HB: R^2^ = 0.31, *p* = 0.010; BB: R^2^ = 0.24, *p* = 0.286).

BB presented a parabolic curve that suggested a threshold in BMI separating players into groups: with lower BMI receiving constantly worse results in COD, and higher BMI receiving better results (Figure 3). The curve for HB was a rather inverted L-shape which has worse results of CODS together with increasing the BMI up to the maximum value and constant plateau beyond the maximum. The calculated thresholds of the BMI were 23.15 for BB and 25.45 for HB.

**Figure 4:**
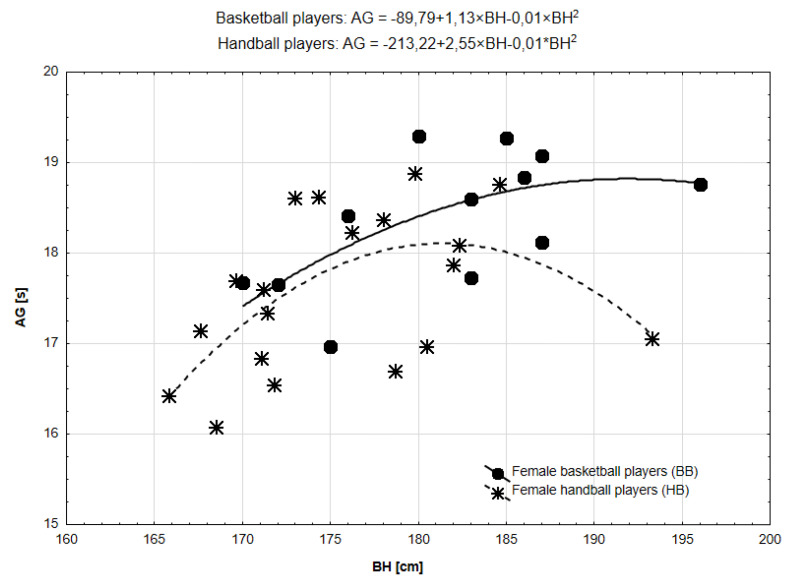
Functional forms of the relationships between BH and AG in BB and HB.

Taking into account the relationship between body height and AG, the forms of the relationships mirrored the functional forms for CODS (Figure 4). The slight difference was that the line for BB was a rather inverted L-shape more similar to the straight line and better drawn by first-degree function (R^2^ = 0.34, *p* = 0.046), while for HB it was a typical parabolic curve (R^2^ = 0.35, *p* = 0.029). Peak values were: BB—191.95 cm; HB—182.23 cm.

**Figure 5:**
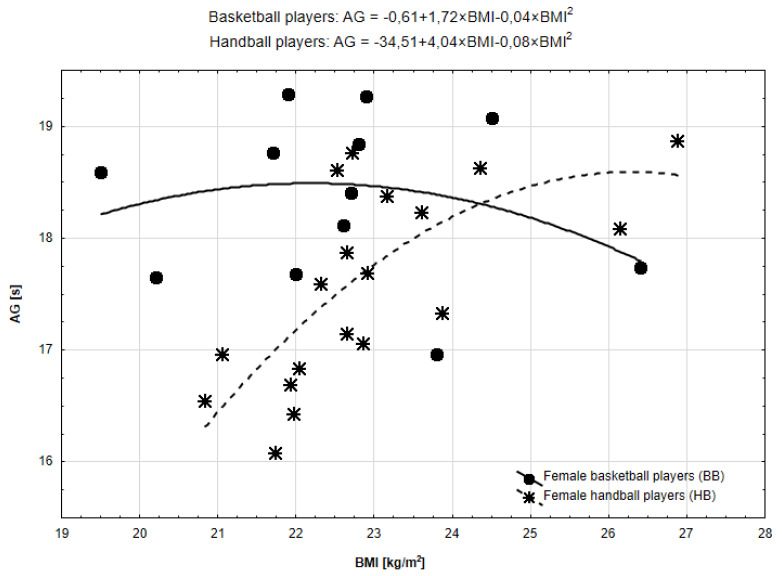
Functional forms of the relationships between BMI and AG in BB and HB.

Taking into account the relationship between BMI and AG, in the case of BB, both models were poor and insignificant (Figure 5). In the case of the HB, the parabolic model was better fitted (R^2^ = 0.51, *p* = 0.003). The scatterplot drawn for the best function mirrored Figure 3 (relationship between CODS and BMI). Peak values were: BB—22.20; HB—26.27.

## 4. Discussion

The main aim of the study was to assess the functional form of the relationship between BH and BMI and motor ability (CODS and AG) in female BB and HB. The hypothesis put forward in some of the relationships between IV and DV was supported. Statistically significant regression models were found in female handball players between BH vs. CODS, BH vs. AG, BMI vs. CODS and BMI vs. AG. On the other hand, in basketball players, only a significant relationship was noticed between BH and AG. The present contribution demonstrated that the functional forms between BH or BMI and COD or AG differ between players from two quite different team sports games. There were the following forms of the relationships observed: (1) constant linear increase, (2) inverted L-shape linear increase with plateau, (3) inverted U-shape increase at the beginning, peak value and decline from peak value together with increase in the anthropometric measurement.

The constant linear increase (similar to a straight line) occurred only in CODS vs. BH relationships among BB (not statistically significant). The taller the BB, the longer the overall test time was for the CODS. A similar principle was observed in the relationship between AG and BH among BB, but it was not a constant line increase. This relationship was L-shaped. It was found in BB that the higher the BH (up to 183 cm), the worse the AG result. On the other hand, athletes over 183 cm obtained similar results in the AG evaluation test. However, these principles noticed in BB were not disclosed in the CODS vs. BH and AG vs. BH relationships in HB. HB were shorter and faster in COD and AG tests than BB. For the CODS and AG results, relationships with the shape of an inverted U were noticed. It was found that the taller the BH (up to 183 cm), the worse the CODS result was. On the other hand, the taller the players are over 183 cm, the better the CODS scores. Similarly, a worsening of the AG scores was observed along with an increase in BH to 182.23 cm. On the other hand, the female athletes with a higher BH than 182.23 cm obtained a shorter total time for the agility test. This proves that tall HB can obtain similar CODS and AG results to female players with a BH of 175 to 183 cm in our study. However, athletes over 183 cm could not achieve a CODS and AG result similar to that of athletes of the lowest height. Study results show that the CODS results are BH dependent and therefore COM dependent. This also has a bearing on the results of AG (through its motor component) [28]. Moreover, the similar results of CODS and AG of tall handball players to short players may confirm the complexity of these motor abilities. Motor deficiencies and differences in the height of COM can be compensated for by improving the technique of change of directions or making decisions [34,35]. It is also important to carry out agility, plyometric, or strength training that cause neuronal adaptation, increased intermuscular coordination, and/or increased proprioception [36,37]. Our results can confirm this kind of coach’s actions. However, the noticed occurrence of peak values in the relations of anthropometric and motor measurements is interesting, which today is difficult to interpret in these relations due to the limited (small) number of samples. This should be the goal of further research interests.

An inverted U-shaped relationship was also noticed between CODS vs. BMI and AG v BMI in BB. These relations were not statistically significant. However, in the CODS and BMI relation in HB, we noticed a significant L-shaped relationship. The higher the BMI of the players (up to 25.45), the worse the CODS test result. Additionally, the higher the BMI than the value of 25.45, the more similar the results of the COD test. The importance of BMI on the results of HB is probably related to their H somatotype, i.e., they are all shorter than the tested BH. BH and somatotype are some of the key anthropometric parameters that influence the sports success of team sports athletes [38]. It is suggested in subsequent studies to determine the somatotype of the player, which is currently a limitation of this research. The peak value of BMI = 25.45 is an overweight value [39]. People with a BMI > 25 are likely to have increased body fat. Increasing it in the athlete’s body negatively affects the results of CODS and possibly AG [40,41,42]. However, the interpretation of BMI should be approached with caution. Its value can be influenced by other body components [39]. On the other hand, calculating BMI does not require specialized research equipment and can be easily calculated by any trainer.

The form of the L-shape linear increase was found also between BMI vs. AG in HB. Agility results worsened in parallel with increasing BMI (up to 26.27). On the other hand, above this BMI value, the AG results improved. The results partially confirm the importance of the perceptual-cognitive component of agility and the results of other studies that found that people with a higher BMI react significantly slower than those with a lower BMI [43,44]. Improving agility results in people with high BMI may be caused by other factors influencing the improvement of agility tasks, such as sports experience, COD technique, or speed of making decisions.

Generally, squared functions better explained the relationship between anthropometric measurement and functional features (R^2^ was higher for those functions than for linear ones). However, there were differences between BB and HB. Models for BB were on a lower level of goodness of fit compared to HB. The best fit was received for the relationship between BMI and AG in HB (squared function, R^2^ = 0.51, *p* = 0.003).

This proves that when analyzing elite athletes, it is not possible to unequivocally determine the linearity of the relationship between anthropometric and agility parameters. Differences in motor development caused by various biological, training, and social factors are eliminated through a properly prepared training process [45]. Often even referring to the individual work of coaches with the player [46]. Therefore, elite players who are taller or have a higher BMI may achieve similar results in motor tests or during the game compared to other athletes.

To the best of the authors’ knowledge, this is the first study that presents the functional forms of the relationship of BH, BMI, CODS, and AG in team sports games. In addition, the study analyzed the relationship between professional players of two different types of sports. These results could be included in a database against which other adult female athletes can be compared. Further, the present study determined the relationship between the anthropometric and motor skills parameters in an agility task based on a uniform ‘stop-and-go’ scenario.

Some limitations are present in this study. Firstly, the small number of the participants limited the statistical approach. There were not enough people to conduct higher-term regressions. Secondly, all analyses were based on cross-sectional data. Third, the researchers analyzed the results without taking into account the players’ playing positions. Numerous studies indicate that the results of motor tests and anthropometric measurements differ depending on the position of the game [47,48]. This should be taken into account in future research.

## 5. Conclusions

The hypotheses put forward in the study (BH and BMI might be significantly associated with results of CODS and AG; the relationships between anthropometric parameters and the results of motor ability might be curvilinear) were partially confirmed. Our results confirmed the relationship between both anthropometric measurements (BH and BMI) and the results of both motor tests (COS and AG) only in female handball players. In BB, a significant relationship was noted only between BH and AG. This contribution, which attempts to identify the shape of the relationships between BH or BMI and CODS or AG, finds that the functional form of the relationship is curvilinear rather than linear (model with the second term of the anthropometric measurement better explained the relationship with CODS or AG). However, detailed analysis showed that the functional form of the relationship is related to team sport discipline. Models for HB were generally better fitted than for BB. Two curvilinear functional forms of the relationship were identified: inverted L-shape (which means that there is a plateau in body height or BMI for CODS or AG) and inverted U-shape (which means the peak value of the BH or BMI which is the threshold for the value of the anthropometric measurement when the progressive effect in functional feature starts). Future studies should focus on searching for causal relationships between morphological and functional features using other explanatory variables, e.g., pole position.

## Figures and Tables

**Table 1:** Descriptive statistics and independent samples *t*-Student test results between types of sport (*t*-value, *p*-value).

Variables	Elite Female Basketball Players				Elite Female Handball Players				*t*	*p*
	Mean	−95CI	+95CI	SD	Mean	−95CI	+95CI	SD		
BH	181.67	176.96	186.38	7.41	175.77	172.44	179.11	6.93	2.25	0.033 *
BMI	22.58	21.42	23.74	1.83	22.96	22.22	23.69	1.53	−0.61	0.544
CODS	15.92	15.57	16.26	0.55	15.31	14.89	15.73	0.87	2.14	0.041 *
AG	18.37	17.90	18.84	0.74	17.57	17.15	17.99	0.87	2.63	0.014 *

* *p* < 0.05, BH—body height, BMI—body mass index, CODS—change of direction speed, AG—agility.

**Table 2:** Regression results for the entire sample (*p*-value for R^2^).

IV	Athletes	Statistic Parameters	DV			
			BH		BMI	
			Function		Function	
			F1	F2	F1	F2
CODS	BB	x	0.02	0.29	0.03	2.67
		x^2^		−0.001		−0.06
		Constant	10.67	13.71	15.20	−14.84
		R^2^	0.15	0.15	0.01	0.24
		*p*	0.209	0.456	0.742	0.286
	HB	x	0.05	1.92	0.31	5.06
		x^2^		−0.01		−0.01
		Constant	5.67	−160.66	8.04	−48.26
		R^2^	0.19	0.32	0.31	0.42
		*p*	0.061	0.045 *	0.010 *	0.012 *
AG	BB	x	0.05	1.13	−0.05	1.72
		x^2^		−0.003		−0.04
		Constant	7.77	−89.78	19.56	−0.61
		R^2^	0.34	0.40	0.02	0.07
		*p*	0.046 *	0.097	0.685	0.707
	HB	x	0.04	2.55	0.37	4.04
		x^2^		−0.007		−0.08
		Constant	9.91	−213.21	8.96	−34.51
		R^2^	0.12	0.35	0.43	0.51
		*p*	0.140	0.029 *	0.002*	0.003 *

* *p* < 0.05, x—coefficient of the anthropometric measurement in regression function, x^2^—squared coefficients of the anthropometric measurement in regression function, DV—dependent variables, IV—independent variables, CODS—change of direction speed, AG—agility, BH—body height, BMI—body mass index, BB—elite female basketball players, HB—elite female handball players, F1—linear form (simple linear regression), F2—second degree function (binomial curvilinear regression).

## Data Availability

Data of body height, CODS, and RA for groups of basketball and handball players presented in this work were registered in the AZON repository: https://deponuj.azon.e-science.pl/entry/57923/detailredirect (accessed on 18 June 2021).
